# Static Hydrophobic Cuprous Oxide Surface Fabricated via One-Step Laser-Induced Oxidation of a Copper Substrate

**DOI:** 10.3390/mi14010185

**Published:** 2023-01-11

**Authors:** Xi Yu, Yoshiki Tanaka, Tomoki Kakiuchi, Takafumi Ishida, Koh Saitoh, Fumihiro Itoigawa, Makoto Kuwahara, Shingo Ono

**Affiliations:** 1 Institute of Materials and Systems for Sustainability, Nagoya University, Nagoya 464-8603, Japan; 2 Department of Engineering, Nagoya Institute of Technology, Nagoya 466-8555, Japan; 3 Graduate School of Engineering, Nagoya University, Nagoya 464-8603, Japan

**Keywords:** femtosecond laser processing, surface modification, hydrophobic surface

## Abstract

In this study, we developed a one-step method for fabricating hydrophobic surfaces on copper (Cu) substrates. Cuprous oxide (Cu_2_O) with low free energy was successfully formed after low-fluence laser direct irradiation. The formation of Cu_2_O enhanced the hydrophobicity of the Cu substrate surface, and the contact angle linearly increased with the proportion of Cu_2_O. The Cu_2_O fabricated by low-fluence laser treatment showed the same crystal plane orientation as the pristine Cu substrate, implying an epitaxial growth of Cu_2_O on a Cu substrate.

## 1. Introduction

Cuprous oxide (Cu_2_O) and copper oxide (CuO) have been investigated for photovoltaic applications [1,2,3,4,5] owing to their feasible bandgap (1.9–2.2 and 1.2–1.7 eV, respectively) corresponding to the visible region. Cu_2_O is also an emerging photocathode for water splitting [6,7,8]. However, there is still a need to develop low-cost methods for preparing high-quality and large-scale crystals of these promising materials [9,10,11,12,13,14]. It is more feasible to fabricate copper oxide complex thin films, which have been achieved on copper (Cu) layers via thermal oxidation for solar cells [15] and laser processing for photodetectors and glucose sensing [16,17]. Laser-treated Cu-substrate surfaces with controllable wettability have also been reported. Long et al. treated laser-fabricated microstructures with CF_3_(CF_2_)_7_CF_2_CF_2_−Si−(OCH_3_)_3_ methanol [18], triethoxyoctylsilane [19], and fluoroalkylsilane [20] to reduce their surface free energy. Pan et al. soaked laser-textured microstructures in a stearic acid solution at ambient temperature for 60 min and dried them in an oven (60 °C) for 10 min to achieve low-adhesive features [21]. He et al. treated laser-fabricated microstructures with ethanol-assisted low-temperature annealing [22], and Ma et al. heated similar structures [23] to transform hydrophilic CuO to hydrophobic Cu_2_O and form hydrophobic organics. In these studies, the wettability and morphology of laser-fabricated microstructures have been extensively studied, and postprocessing was needed to add the hydrophobic base to the surface. It is also reported that a lower oxygen amount of copper-oxidation surface is associated with lower surface energy, leading to an increase in hydrophobicity [24]. Cu_2_O surfaces have been fabricated by chemical bath deposition [24] and electrodeposition [25,26] for achieving a hydrophobic surface. In our previous study, we developed a one-step patterning oxidation method for Cu surfaces via femtosecond laser irradiation in the atmosphere [27]. The high peak fluence due to the ultrashort pulse duration promoted the chemical reaction between Cu and oxygen. To avoid the formation of structures in the micrometer order, the samples were treated under low average fluence to suppress laser ablation. Using this one-step surface modification method, we fabricated a Cu_2_O layer with a thickness of less than 30 nm on the surface of a Cu substrate. Without any postprocessing, the one-step laser-treated surface showed enhanced hydrophobic features owing to the formation of low-free-energy Cu_2_O. In addition to improving the useful time of Cu-based machine parts due to the self-cleaning feature of hydrophobic surfaces, this one-step laser-induced Cu_2_O surface also showed some potential applications, such as biocides [28], photovoltaic devices [29], and photocatalysts [6].

## 2. Materials and Methods

To determine the laser processing threshold of Cu substrates (113513, Nilaco, Japan), femtosecond laser pulses (wavelength: 1030 nm, pulse width: 700 fs, repetition rate: 100 kHz, PHAROS PH1–10, Light Conversion, Vilnius, Lithuania) with pulse energy of 5–60 μJ were focused and irradiated on a Cu substrate surface. The area of the single-pulse irradiated spots was measured by confocal laser scanning microscopy (CLSM, LEXT ILS4100, Olympus, Tokyo, Japan) to calculate the ablation threshold energy of Cu. Before fabricating hydrophobic samples with a large area, two 0.5 mm × 0.5 mm samples on Cu substrates were fabricated with low laser fluence. Their surface morphologies were evaluated by scanning electron microscopy (SEM, JSM-5600, JEOL, Tokyo, Japan), and their surface oxidation state was confirmed by Raman spectra (Nanofinder FLEX, TII, Tokyo, Japan). The thickness of oxidation layers was evaluated by scanning transmission electron microscopy and energy dispersive X-ray spectrometry (STEM-EDX, JEM-2100F/HK, JEOL, Tokyo, Japan). Based on the initial experimental results, samples with a size of 4 mm × 4 mm were fabricated by low-fluence laser irradiation. The surface roughness was measured by an atomic force microscope (AFM, JSPM-5200TM, JEOL, Tokyo, Japan). X-ray patterns (XRD, SmartLab, Rigaku, Tokyo, Japan), and the contact angle (CA, Smart Contact PRO 100, Excimer Inc., Yokohama, Japan) of these samples were also measured to investigate the relationship between their surface chemical composition and hydrophobicity. 

## 3. Results and Discussion

**Figure 1:**
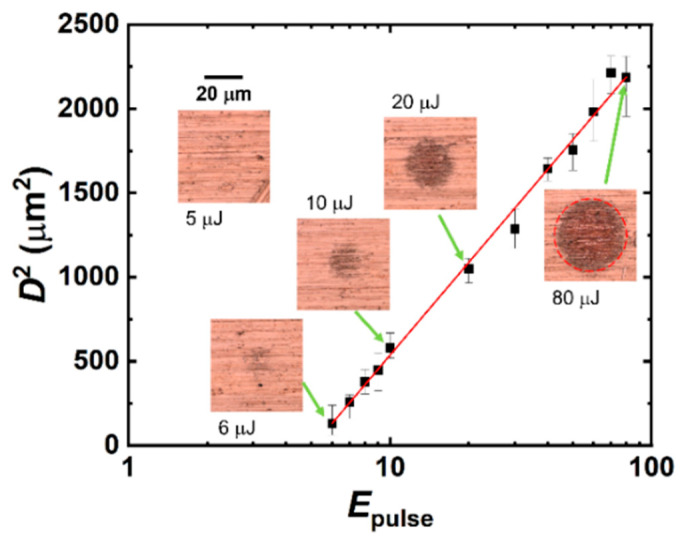
Relationship between the laser pulse energy (*E*_pulse_) and the spot diameter square of the discolored area (*D*^2^). The discolored area is shown in the inserted CLSM images.

**Figure 2:**
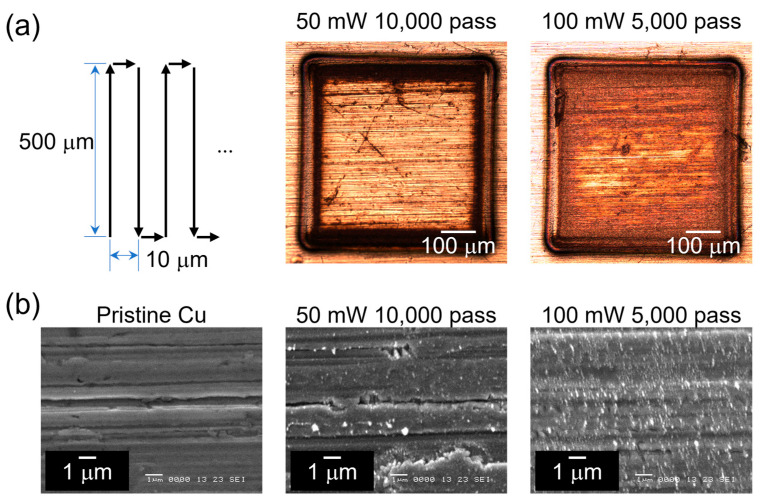
(**a**) Laser scanning pattern and images of the laser-irradiated samples. (**b**) SEM images of pristine, 50 mW-, and 100 mW-irradiated samples.

Figure 1 shows the spot diameter square (*D*^2^) as a function of pulse energy (*E*_pulse_). The measured *D*^2^ was fitted to $D^{2}=2\omega_{0}^{2}\ln\frac{E_{\mathrm{pulse}}}{E_{\mathrm{th}}}$, where *E*_th_ is the ablation threshold energy, and the slope of the fitting is the beam radius at the surface (*ω*_0_) [30]. According to the fitting result, *E*_th_ is 5.1 μJ, and *ω*_0_ is 19.9 μm. When the pulse energy is less than *E*_th_ (5.1 μJ), the irradiated area shows no visible discoloration in the CLSM image. The 0.5 mm × 0.5 mm samples were irradiated by femtosecond laser pulses with an *E*_pulse_ of 0.5 μJ (50 mW at a 100 kHz repetition rate) and 1 μJ (100 mW at a 100 kHz repetition rate), which are much lower than *E*_th_. Laser-induced plasma was not observed during the laser irradiation. The scanning patterns and their images are shown in Figure 2a. The laser beam was guided using a galvanometer scanner head, and the scan speed was 1000 mm/s with respect to the substrate surface. The scanning line space is 10 μm. The 100 mW-irradiated area showed a more pronounced discoloration than the 50 mW-irradiated area, even though they were treated with the same total input energy. Figure 2b shows the SEM image of the pristine and laser-irradiated areas. Instead of the general microstructure fabricated by laser ablation, the low-fluence-treated surfaces showed nanoprotuberances. Mori et al. reported similar nanoprotuberances on a plasma-treated Cu surface, and they also confirmed the formation of the Cu^1+^ on the Cu surface with these nanostructures [31]. The pristine Cu showed brushing trenches on the surface, and these trenches remained after the low-fluence irradiation. 

**Figure 3:**
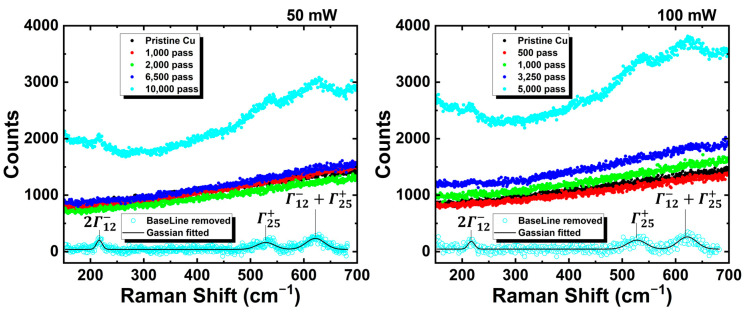
Raman spectra of the low-fluence laser-irradiated Cu substrate surface.

**Figure 4:**
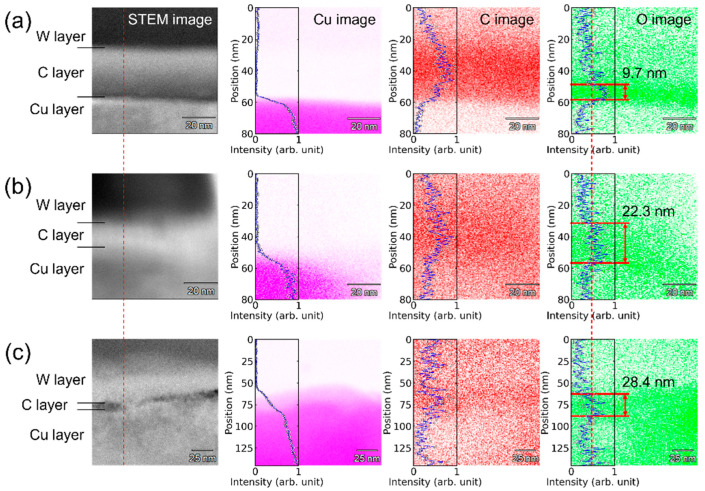
STEM images (cross-sectional view), Cu, C, and O mappings of (**a**) pristine, (**b**) 50 mW- irradiated, and (**c**) 100 mW-irradiated Cu surfaces. The red dotted line in the O image indicate the half maximum of the normalized intensity.

Figure 3 shows the Raman spectra of the low-fluence-treated Cu surface. The solid dots indicate the measured results. The hollow dots show the baseline-removed Raman spectrum [32], and the black line is Gaussian-fitted results from the baseline-removed Raman spectrum. Three peaks were observed for the 10,000- and 5000-pass-treated samples. Based on the fitting results, the three peaks at 216, 524, and 625 cm^−1^ are attributed to Cu_2_O for the second-order overtone of the silent $\Gamma_{12}^{-}$ mode, the Raman-active $\Gamma_{25}^{+}$ mode, and the combination mode of $\Gamma_{12}^{-}+\Gamma_{25}^{+}$ [33,34,35,36,37], respectively. To confirm the crystallinity of the laser-irradiated area, we cross-sectionally picked up thin specimens from the irradiated center of the 500 μm × 500 μm samples using the focused ion beam (FIB) technique (Hitachi FB2100, Japan) and evaluated them by STEM-EDX. It is difficult to identify the type of oxidation based on the measured lattice space because those of Cu, Cu_2_O, and CuO are very close. EDX element mapping showed the thickness of the oxidized layer (Figure 4). Based on the mapping results, oxygen (O) layers were sandwiched between the carbon (C) and Cu layers, indicating that the oxygen was on the Cu surface before the FIB processing. A 9.7 nm-thick O layer due to natural oxidation was observed on the surface of the pristine Cu substrate. Its thickness increased by more than two times for the 50 mW-irradiated surface and three times on the surface of the 100 mW-irradiated surface. The thickness of the O layer was facilitated by laser irradiation, and higher power resulted in thicker oxidation layers owing to the longer laser penetration lengths.

Table 1 shows the fabrication conditions and experiment date of laser processing, XRD, and contact angle measurement. Three samples (current samples in Table 1) with a 4 mm × 4 mm area (scanning line space: 10 μm, laser wavelength: 1030 nm, repetition rate: 100 kHz) were fabricated to evaluate the relationship between their wettability and surface oxidation conditions, and a sample from our previous study (previous sample in Table 1, fabricated using another laser with a wavelength of 1045 nm, a pulse duration of 700 fs, and a repetition rate of 100 kHz at 25 mW) [27] was also evaluated. 

**Figure 5:**
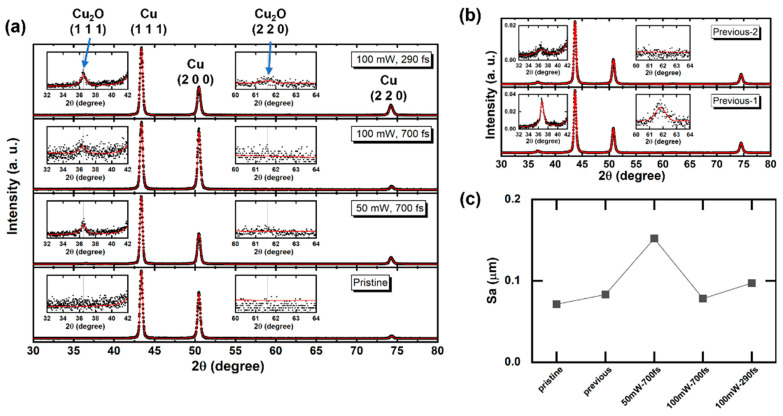
(**a**) XRD patterns of current samples and a pristine Cu substrate. (**b**) XRD patterns of the previous sample measured on different dates with a four-year interval. (**c**) Surface roughness of these samples and a pristine Cu substrate.

Figure 5a,b show the XRD pattern of the laser-irradiated Cu surfaces. The black dots indicate the measured data, and the red lines show the PseudoVoigt function fitting results [38,39]. Compared with the pristine Cu surface, a peak attributed to Cu_2_O (1 1 1) with a lattice spacing (*d*) of 2.09 Å was observed for all laser-irradiated samples. In Figure 5b, the Previous-1 and Previous-2 are XRD patterns with different measurement dates for the same sample from our previous work. Additionally, the Previous-1 XRD pattern shows a peak attributed to Cu_2_O (2 2 0) (*d*: 1.28 Å). The orientation of (2 2 0) was also observed for the pristine Cu substate. Compared with the monoclinic structure of CuO, Cu_2_O has the same cubic structure as Cu, which may promote the growth of Cu_2_O on a Cu substrate. Barton et al. [40] reported epitaxial electrodeposition of Cu_2_O on single-crystal Cu owing to the similar cubic cell structures of Cu_2_O and Cu. They also reported that an epitaxial-grown Cu_2_O (2 0 0) (*d*: 1.81 Å) thin film on Cu (2 0 0) (*d*: 2.13 Å) has a thickness limitation. On a Cu (2 0 0) substrate, Cu_2_O (1 1 1) first grows instead of Cu_2_O (2 0 0), and Cu_2_O (2 0 0) appears when the film is thicker than 360 nm. Previous-2 showed a weaker Cu_2_O (1 1 1) peak than that of Previous-1 and no Cu_2_O (2 2 0) peak. This weakening of the Cu_2_O peak can be attributed to the natural oxidation of Cu_2_O to CuO, and the increased proportion of CuO was too low to be detected by XRD. Kwon et al. [16] reported that CuO was formed in the laser ablation center; in contrast, Cu_2_O was formed in the surrounding where it has lower energy density, i.e., laser fluence, than that of the center due to the Gaussian distribution of fluence. In the laser-irradiated spot, the fluence distribution determines the temperature distribution, thus determining the type of formed oxide. Lower fluence is associated with lower temperature, which facilitates the formation of Cu_2_O. Consequently, the low-fluence treatment in this study favored Cu_2_O formation. The arithmetical mean height (Sa) was used to evaluate the surface roughness as shown in Figure 5c. Sa increased less than 0.1 μm when irradiated by low laser fluence.

**Figure 6:**
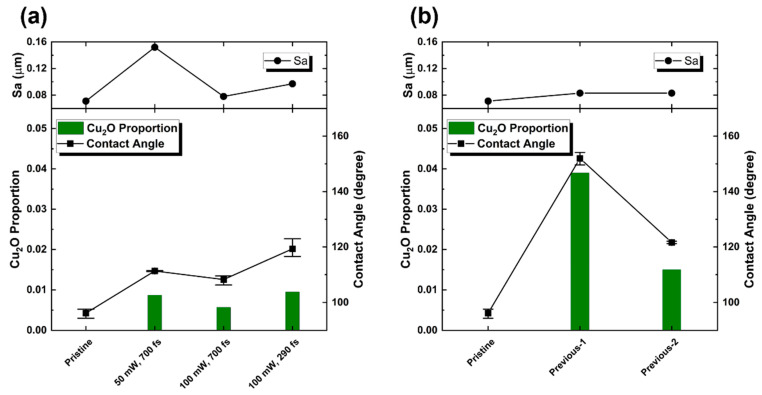
Contact angle, Sa, and Cu_2_O proportion of the pristine and laser-irradiated samples. (**a**) 4 mm × 4 mm samples. (**b**) Previous sample with different experiment dates of CA and XRD. The droplets used for the CA measurement have a volume of 3 μL. The CAs were automatically confirmed by pre-installed software which can automatically detect the edge of droplets.

**Figure 7:**
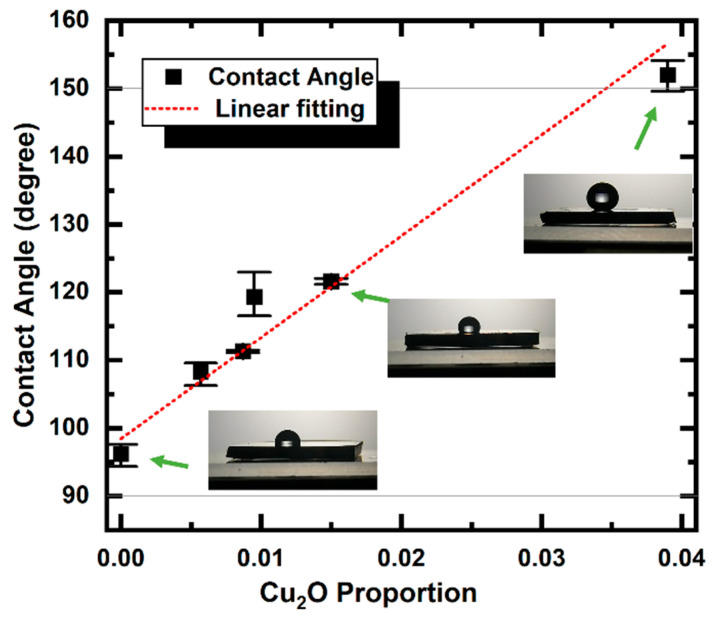
Relationship between Cu_2_O proportion and CA.

Figure 6 plots the CA, Sa, and Cu_2_O proportion of the pristine and laser-irradiated samples. The Cu_2_O proportion was obtained from the ratio of the peak area (PseudoVoigt function fitting) attributed to Cu_2_O to that of Cu. In Figure 6a, the Sa increased by less than 0.1 μm, and in Figure 6b the Sa is almost kept the same for the pristine and the previous sample. The low-fluence laser-treated samples did not change too much in the surface roughness, which cannot significantly change the CA [25,41]. On the contrary, the proportion of Cu_2_O showed a positive correlation to the CAs. These results suggest that the surface free energy will be dominant to the static CA when the surface roughness insignificantly changes. Figure 7 shows the relationship between the Cu_2_O proportion and CA of the samples. CA increased linearly with the Cu_2_O proportion. The oxidation of metal copper may mitigate the surface free energy of pristine Cu, consequently enhancing the surface hydrophobicity. Meanwhile, a lower amount of oxygen results in lower surface energy [24], and Cu_2_O facets show different surface energies: *E*_111_ < *E*_110_ < *E*_100_ [42], i.e., Cu_2_O (1 1 1) exhibits higher hydrophobicity than other faces. In the report of Kwon et al. [43], Cu_2_O showed the largest CA than that of Cu and CuO due to their different interparticle bonds [44]. These results suggest that, besides the surface structure in micro-order, the chemical composition is an important factor determining the wettability of the surface. In addition, hydrophobicity is controllable by this simple method because surface chemical compositions can be controlled by varying the laser conditions to create different reaction conditions and atmospheres.

## 4. Conclusions

In conclusion, via one-step laser irradiation, we fabricated hydrophobic surfaces on Cu substrates owing to the formation of low-free-energy Cu_2_O. The temperature of a laser-irradiated area could be adjusted by controlling the laser fluence, which determines the chemical composition of the copper oxides. The CA of the Cu surface exhibited enhanced hydrophobicity after the simple one-step process, showing a linear increase in the Cu_2_O proportion. The laser-induced Cu_2_O showed the same plane orientation as the Cu substrate owing to their similar cubic cell structures, implying an epitaxial growth of Cu_2_O on Cu. This simple one-step technique can promote the application of laser-induced functional surfaces and demonstrates the potential of laser-assisted epitaxial growth of metal oxides via direct laser irradiation in the atmosphere.

## Figures and Tables

**Table 1:** The details of fabrication conditions and experiment date. These samples were kept in sample cases which were stored in a desiccator, and a clean tweezer was used to pick these samples when they needed to be moved.

Samples	Current samples			Previous sample	
	50 mW, 700 fs	100 mW, 700 fs	100 mW, 290 fs	Previous-1	Previous-2
laser wavelength	1030 nm	1030 nm	1030 nm	1045 nm	
pulse duration	700 fs	700 fs	290 fs	700 fs	
pulse energy	0.5 μJ	1 μJ	1 μJ	0.25 μJ	
repetition rate	100 kHz	100 kHz	100 kHz	100 kHz	
scan speed	1 m/s	1 m/s	1 m/s	1.53 m/s	
scan line space	10 μm	10 μm	10 μm	22.5 μm	
fabricated date	5 April 2022	31 March 2022	18 March 2022	10 January 2018	
XRD date	12 May 2022	12 May 2022	12 May 2022	15 January 2018	12 May 2022
CA date	23–25 May 2022	23–25 May 2022	23–25 May 2022	20 July 2020	23–25 May 2022
Sa date	27 December 2022	27 December 2022	27 December 2022	27 December 2022	

## Data Availability

The data that support the findings of this study are available from the corresponding author upon reasonable request.
